# Post-irradiation physicochemical features of polymer composite for the removal of Co(II) and Nd(III) from aqueous solutions

**DOI:** 10.1007/s11356-022-22862-8

**Published:** 2022-09-13

**Authors:** Eman H. El-Masry, Tarek M. Mohamed, Sayed S. Metwally

**Affiliations:** 1 Egyptian Atomic Energy Authority, Hot Labs. Center, Cairo, Post Code 13759 Egypt; 2 Egyptian Atomic Energy Authority, National Centre for Radiation Research and Technology, Cairo, Egypt

**Keywords:** Gamma irradiation polymerization, Sorption, Wastewater, Cobalt and neodymium ions

## Abstract

The scientific impact of this work is the protection of the environment from hazardous pollutants. Gamma irradiation was employed for the preparation of a new composite polymer by irradiating a mixture containing polyvinyl pyrrolidone (PVP), hydroxyethyl methacrylate (HEMA), and tannic acid (TA) to produce PVP–HEMA–TA. The sorption efficiency and capacity of PVP–HEMA–TA were evaluated by studying some factors affecting the sorption of Nd(III) and Co(II) from aqueous solutions. The results demonstrated that the maximum uptake was 92.4 and 75.3% for Nd(III) and Co(II), respectively. From the kinetic studies, the pseudo-second-order equation could better fit the data than the pseudo-first-order for the sorption of both ions. The sorption isotherm investigations illustrated that the Langmuir equation fits the gained data better than Freundlich equation. The Langmuir capacity was 64.5 and 60.8 mg/g for neodymium and cobalt ions, respectively. The applicability of Langmuir equation is strong evidence that the process is limited by a chemisorption mechanism. Findings of the work highlight the potential utilization of PVP–HEMA–TA as an effective and recyclable material for the elimination of Nd(III) and Co(II) from the aqueous phase.

## Introduction

Several hazardous ions are present in wastewater including Cs^+^, Eu^3+^, Sr^2+^, Nd^3+^, and Co^2+^, and these should be treated with care and safety using adequate approaches (Metwally et al. 2020). Cobalt is a toxic ion that presents not only in nuclear waste but also in industrial wastewater (Quynh et al. 2021). ^60^Co is an activation product and widely utilized in different fields including sterilization, radiotherapy, and industrial radiography (Vivas and Cho 2021; Metwally et al. 2019). Although its concentration is particularly low, it simply aggregates in the human body. High levels of cobalt in the body will cause varied health troubles as diarrhea, lung irritations, low blood pressure, bone defects, and paralysis (Ghaly et al. 2018). Hence, the waste containing cobalt ions should be treated.

According to the USGS (United States Geological Survey), lanthanide’s global production grows from 124 tons in 2010 to 167 tons in 2018 (USEPA 2012). Despite the economic benefit of lanthanides, the growth in their utilization in several areas led to an increase in their waste. Toxicological investigations state that lanthanides have a significant reverse impact on biota (Metwally et al. 2018). Hence, the separation and recovery of lanthanides from radioactive and industrial wastes are essential both economically and environmentally. Neodymium is utilized in various electronic devices. It is employed as a burn-up monitor for estimating the efficiency of nuclear fuel (Mohamed et al. 2017).

In the view of current scenario of constant industrial expansion and, consequently, the growth in the pollution of water bodies by the discharge of contaminated effluents, different techniques for the capture of contaminants were increasingly explored including adsorption (Hamed et al. 2019; Ahmed et al. 2020), ion exchange (Abdel Rahman et al. 2018), impregnation (Rizk et al. 2018; Metwally and Attallah 2019), biosorption (Metwally et al. 2017), membrane (Hegazy et al. 2006), or mixed techniques.

Recently, polymer composites gain great attention resulting from their relatively low cost and high sorption capacity (Wang et al. 2010; Mohammed et al. 2020). The composite polymers based on the copolymer mixture have great swelling relative to their single monomer (Kaith et al. 2012; Ghobashy and Mohamed 2019). Therefore, the polymer composite is employed in wastewater treatment.

In this work, a polymer composite was produced using gamma irradiation. The physicochemical features of the irradiated polymer were elaborated. Some factors influencing the swelling and gelation percentages of the composite were performed including the impact of tannic acid concentration, irradiation dose, and contact time. Also, the sorption efficiency of the composite for the removal of Nd(III) and Co(II) ions from the liquid phase was established.

## Experimental

### Chemicals

Hydroxyethyl methacrylate (HEMA) was purchased from Sigma-Aldrich. Polyvinyl pyrrolidone (PVP) was gained from Qualikems Fine Chem. Pvt Ltd. Tannic acid (TA) was purchased from Pioneers for Chemicals Co. (Piochem). Cobalt and neodymium nitrates were obtained from Merck. NH_4_OH and HNO_3_ were obtained from Winlab. All chemicals are of ultrapure grade.

### Preparation of the polymer

In a 200-mL beaker, 3.0 g of polyvinyl pyrrolidone (PVP) was dissolved in 70 mL of hot water; after that, 20 mL hydroxyethyl methacrylate(HEMA) was added to the beaker with stirring. Different concentrations of TA (0.5–5%) were added to the mixture with continuous stirring. Finally, the mixture was poured into a glass bottle then exposed to different doses (5–30 kGy) of gamma radiation. After irradiation, the solidified composite was washed three times with 100 mL acetone, dried in an air oven at 60°C, and grinded into small pieces to produce PVP–HEMA–TA.

### Characterization of PVP–HEMA–TA composite

The prepared PVP–HEMA–TA composite was characterized before and after the sorption by Fourier-transform infrared spectroscopy (FT-IR) (ALPHA II; Bruker, Germany). Scanning-electron-microscope photos with EDX mapping of PVP–HEMA–TA composite before and after sorption of the metal ions were gained by JEOL-JSM 6510 LA (Japan). Also, the crystal structure of PVP–HEMA–TA composite was explored by an X-ray powder diffractometer (Philips Analytical PW-1710) equipped with Cu Kα radiation at a scanning speed of 2°/min from 10 to 90°, operated at a voltage of 40 kV and applied potential current of 30 mA.

### Factors affecting the swelling and gelation percentages

Some factors were studied to estimate the degree of swelling and gelation percent of the polymer including TA concentration, contact time, and irradiation dose. One milliliter of different concentrations of TA, 0.5–5%, was utilized at 2% PVP, 20% HEMA, and irradiation dose of 20 kGy for preparation of PVP–HEMA–TA. The product was crushed and dried. A weighed sample (*w*_*i*_) was impregnated with hot water at 60°C for 1 h and then dried at 50°C until a constant weight was gained (*w*_*g*_). The gelation percent was computed using Eq. (1). For calculating the water uptake (swelling), 1.0 g of the dried composite, *W*_1_, is soaked in bidistilled water at room temperature; after that, the soaked samples were removed and weighed, *W*_2_, then returned to the water again for repeated reading until constant weight is gained. The swelling of PVP–HEMA–TA composite was computed as illustrated by Eq. (2). The swelling of the sample in water leads to the dissolving of unreacted parts which leads to the removal of homopolymer.

1 $$Gelation,\%=\frac{w_g}{w_i}\times 100$$

2 $$Swelling,\%=\frac{w_2-{w}_1}{w_1}\times 100$$

Also, the contact time (1–12 h) for the preparation of PVP–HEMA–TA was investigated at 2% PVP, 20% HEMA, and 2% TA at the dose of 20 kGy and calculating the swelling percent in each case. The swelling percent was valued by Eq. (2). The effect of irradiation dose of 5–30 kGy on the polymer preparation was also studied by mixing 2% TA, 2% PVP, and 20% HEMA for 3 h. The gelation percent was considered from Eq. (1).

### Sorption investigations

#### Impact of pH

Distribution coefficients of Nd(III) and Co(II) in solutions on PVP–HEMA–TA composite were calculated by the batch technique. PVP–HEMA–TA (0.1 g) was agitated with 10 mL of 200 mg/L of Nd(III) and/or Co(II) ions solution at altered pH values (1.0–6.0) and room temperature for 3.0 h. After equilibration, the mixture was separated then the filtrate was used for determining the ion concentration using an atomic absorption spectrophotometer (Buck Scientific Model 210 VGP, USA). All measurements were approved in duplicates. The distribution coefficient, *K*_d_ (mL/g), and the percent uptake of neodymium and cobalt ions were computed as follows:

3 $${K}_d\left(\frac{mL}{g}\right)=\frac{C_o-{C}_e}{C_e}\times \frac{V}{m}$$

4 $$Uptake\left(\%\right)=\frac{C_o-{C}_e}{C_o}\times 100$$

where *C*_*o*_ and *C*_*e*_ are the original and final concentrations of the ions, respectively, *V* is the sample volume (mL), and *m* is the weight of PVP–HEMA–TA.

#### Kinetic studies

For determining the time interval required to practically access the equilibrium, 50-mL glass bottles each contain 0.1 g of PVP–HEMA–TA and 10 mL of 200 mg/L of Nd(III) and/or Co(II) solutions at optimum pH. The bottles were agitated at the ambient temperature for different times (5–180 min). After centrifugation, the concentration of the ions, *C*_*t*_, was measured in the filtrate. The percent uptake for each was calculated by Eq. (3) after replacing *C*_*e*_ with *C*_*t*_.

#### Equilibrium investigations

Experimentations of batch equilibrium were attained at different temperatures (298–328 K), liquid-to-solid ratio (V/m, 100 mL/g), and different ion concentrations (20–500 mg/L). The mixes were agitated to reach equilibrium. The equilibrium concentration, *C*_*e*_ (mg/L), in the filtrate was measured. The quantity of ion sorbed onto PVP–HEMA–TA composite at equilibrium, *q*_*e*_ (mg/g), was computed as follows:

5 $${q}_e\left(\frac{mg}{g}\right)=\left({C}_o-{C}_e\right)\times \frac{V}{m}$$

### Desorption experimentation

Desorption experimentation of Co(II) and Nd(III) by PVP–HEMA–TA composite was assessed using 0.5 mol/L HNO_3_ at ambient temperature, 25°C. First, the sorption stage was performed by mixing 0.1 g of PVP–HEMA–TA with 10 mL of 200 mg/L cobalt and/or neodymium ions at the optimum pH and agitating for equilibrium. The ion concentration in the filtrate, *C*_*e*_, was computed and the uptake percent was considered by Eq. (3). The ion concentration in the solid phase, *C*_*s*_, was computed from the difference between the initial concentration, *C*_*o*_, and *C*_*e*_. Second, the desorption stage was implemented by agitating the loaded PVP–HEMA–TA with 10 mL of 0.1 mol/L HNO_3_ for equilibrium. The ion concentration in the liquid, *C*_*d*_, was gained. The desorption percent, *D%*, of ions was gained by Eq. (6).

6 $$D\%=\frac{C_d}{C_s}\times 100$$

## Results and discussion

### Characterization of PVP–HEMA–TA composite

**Fig. 1:**
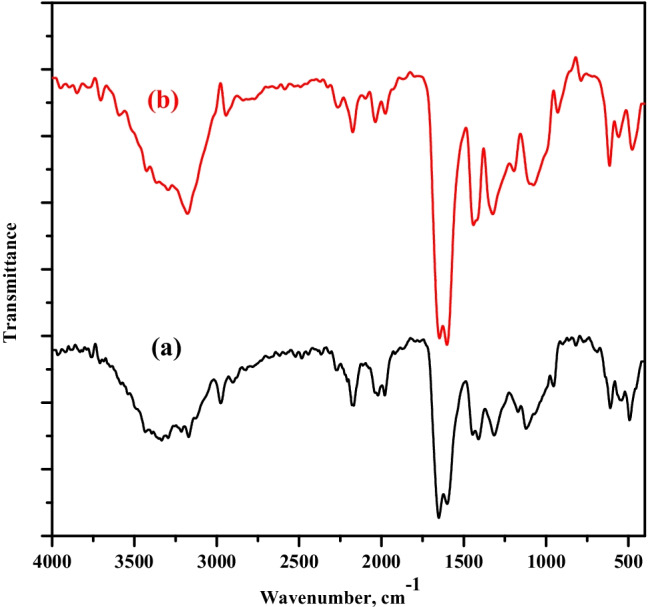
FT-IR spectra for PVP–HEMA–TA composite (**a**) after and (**b**) before sorption

The spectrum of TA is obtained as displayed in Fig. 1 where a wide absorption band can be observed around 3600–3000 cm^−1^ centered at 3270 cm^−1^ (Teguh et al. 2019). This band is characteristic of the hydroxyl groups (OH) and C–H of the aromatic rings. The peak at 2833 cm^−1^ is assigned to C–H of the aliphatic groups. Tannic acid includes aromatic esters related to the carbonyl groups, C=O, stretching which appeared at 1650 cm^−1^ and C–O group which appeared at 1200 cm^−1^. The characteristic peaks of –C–N, −CH_2_, and −CH groups in PVP were observed at 1280, 1450, and 1370 cm^−1^, respectively (Bryaskova et al. 2011). Also, the strong characteristic peak of carbonyl groups, C=O, in PVP and HEMA is gained at 1650 cm^−1^. The hydroxyl group, OH, of HEMA is observed at the wideband around 3000–3600 cm^−1^. The stretching C=C band in HEMA appeared at 1637 cm^−1^ (Belaidi et al. 2015). After the sorption process, the peaks become weaker than that before the sorption; this confirms that the sorption occurs.

**Fig. 2:**
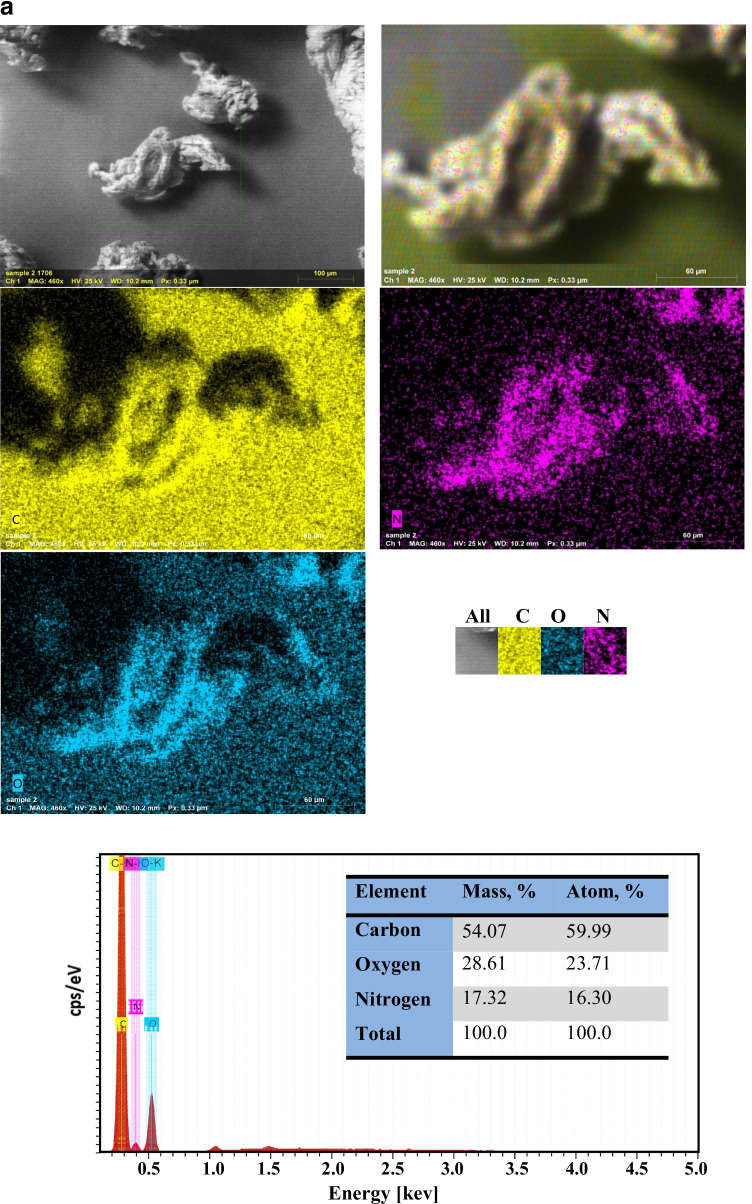

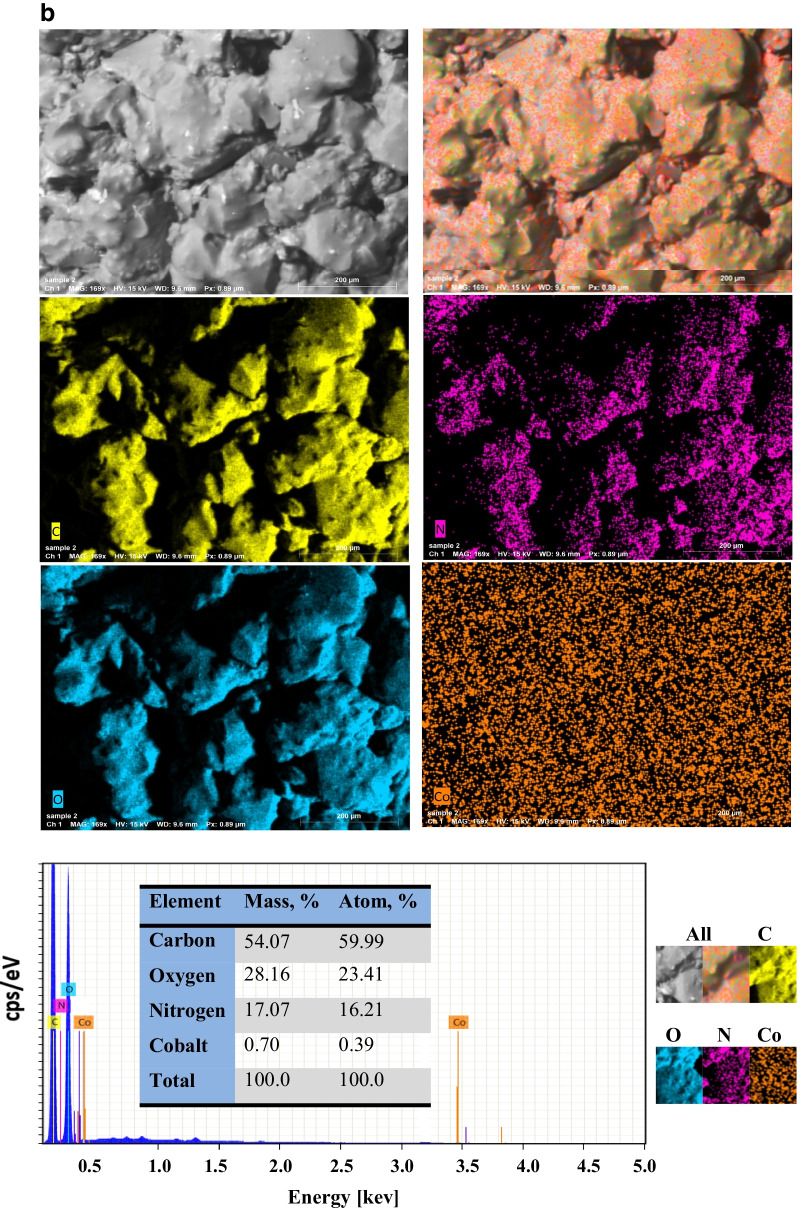
**a** SEM with EDX-mapping of PVP–HEMA–TA before the sorption process. **b** SEM with EDX-mapping of PVP–HEMA–TA after the sorption process

Figure 2 displays SEM with EDX mapping of PVP–HEMA–TA before and after the sorption process, respectively. Figure 2a shows that PVP–HEMA–TA is essentially composed of C, O, and N ions. This was established by EDX which demonstrates the existence of both atoms; carbon is the most abundant ion with a mass percent of 54.07%, whereas oxygen and nitrogen ions are present with 28.61 and 17.32%, respectively. Figure 2b displays the spectroscopic proof for the existence of the sorbed metal ions with PVP–HEMA–TA. This approves the ions’ sorption by the composite polymer.

### Factors influencing the swelling and gelation

**Fig. 3:**
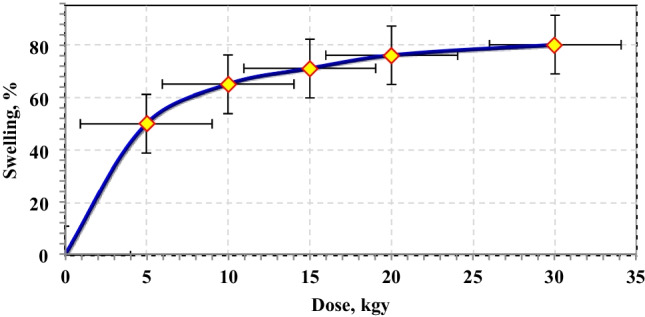
Effect of irradiation dose on the gelation % at 2% TA, 2% PVP, and 20% HEMA

The impact of irradiation dose on the gelation percent of PVP–HEMA–TA was investigated as displayed in Fig. 3. The data illustrated that the gelation percent rises with raising the irradiation dose due to an increase in the free radicals which increases the crosslinking. The optimum irradiation dose was selected at 20 kGy.

**Fig. 4:**
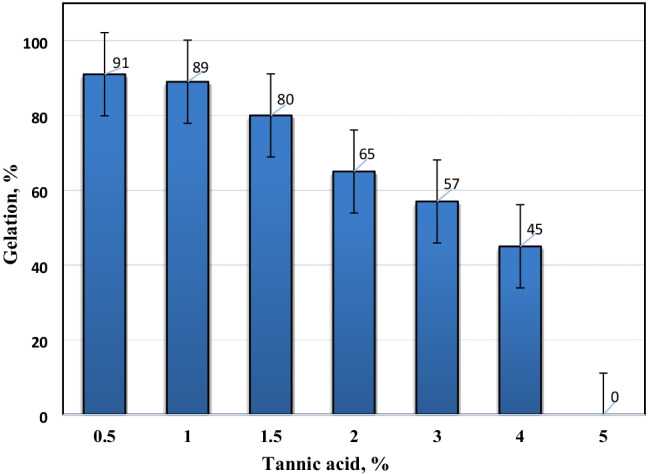
Effect of tannic acid concentration on the gelation % of PVP–HEMA–TA at 2% PVP, 20 kGy, and 20% HEMA

The dependence of gelation percent of PVP–HEMA–TA on TA concentrations is displayed in Fig. 4. It is observed that the gelation percent minimizes with raising TA concentration in the polymer compositions. This may be attributed to the increase in TA concentration that hinders the gamma radiation to reach the polymer, and this decreases the free radicals’ concentration which reduces the crosslinking of the hydrogel structure during the irradiation polymerization process; hence, the gelation percent decreased. The optimum concentration of TA was desired at 2%.

**Fig. 5:**
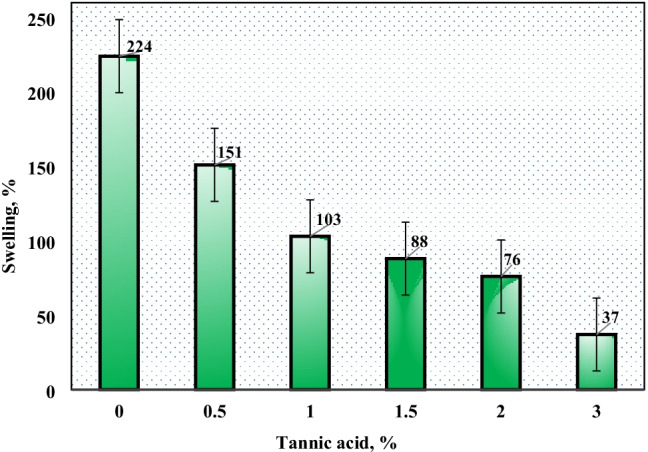
Effect of tannic acid concentration on the water uptake of PVP–HEMA–TA at 2% PVP, 20 kGy, and 20% HEMA

**Fig. 6:**
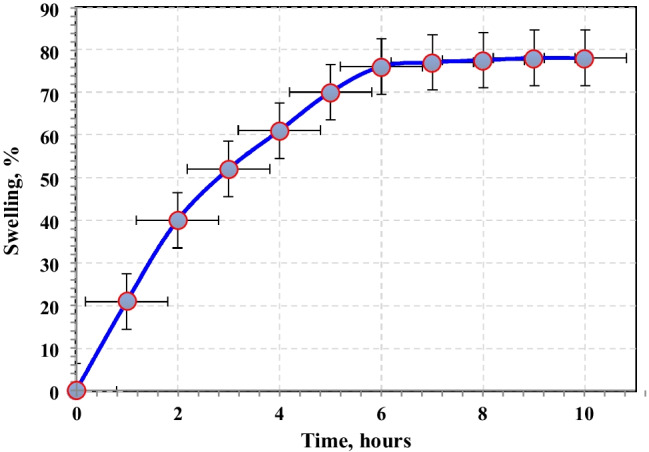
Effect of contact time of preparation on the swelling % at 2% TA, 2% PVP, 20% HEMA, and 20 kGy

The impact of TA concentration on the swelling percent of PVP–HEMA–TA is displayed in Fig. 5. Similarly, the results demonstrated that the swelling percent is inversely proportional to the TA concentration. Also, the progress of water uptake was observed by calculating the swelling percent of the polymer as increasing at selected time intervals as illustrated in Fig. 6. A sharp increase in the swelling percent was gained at the first hour then the swelling percent increases gradually until it becomes constant after 6 h. From these studies, the optimum conditions of irradiation dose, concentration of TA, and HEMA were desired at 20 kGy, 2%, and 20%, respectively.

### Sorption investigations

#### Effect of pH

**Fig. 7:**
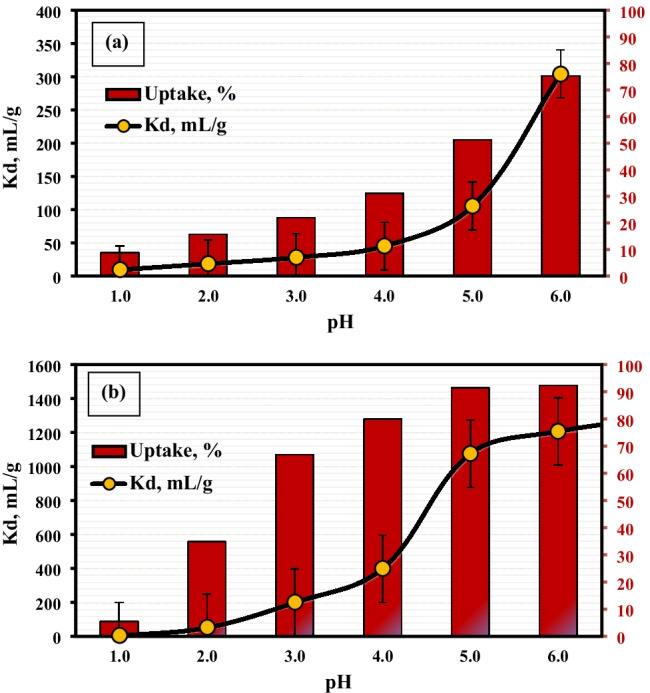
Effect of pH on sorption of 200 mg/L of (**a**) Co^2+^ and (**b**) Nd^3+^ by PVP–HEMA–TA at room temperature and V/m=100 mL/g

The pH is the more significant parameter controlling the sorption process. This is relatively sorbed since hydrogen ions themselves powerfully compete with cations. The pH influence on the capture of Co(II) and Nd(III) ions was performed at pH range of 1.0–6.0; higher pH values were not studied to avoid the precipitation of both ions (Mohamed et al. 2017). The outcomes are displayed in Fig. 7; the data demonstrated that the uptake percent and the distribution coefficient, *K*_*d*_, of both cations rise by raising the pH value. This is due to reduced competition between the proton and the ions. The maximum uptake was 92.4 and 75.3% for Nd^3+^ and Co^2+^, respectively, and the maximum values of *K*_*d*_ are 1207 and 304 mL/g, respectively, as indicated by Fig. 7. Hence, the optimum pH was preferred at pH 5.5, while pH 6.0 was not desired to be sure there is no precipitation.

**Fig. 8:**
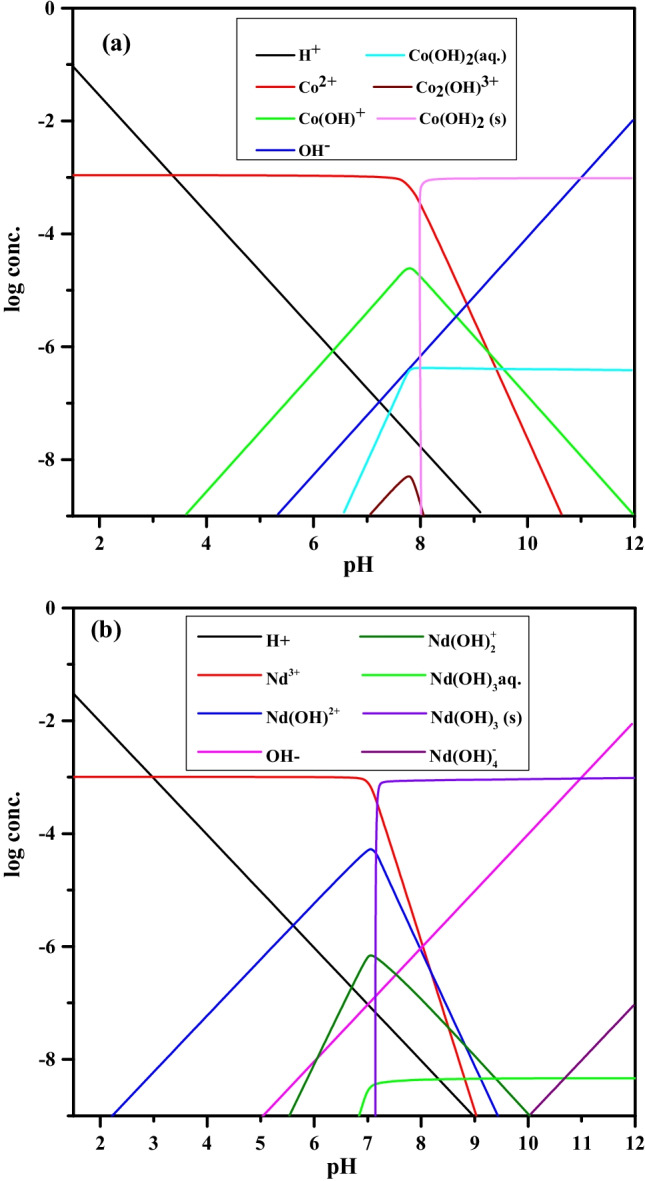
Speciation of (**a**) Co and (**b**) Nd ions at different pH values

The speciation was conducted at pH range 1.5–12 using MEDUSA software (Uigdomenech 2013) and displayed in Fig. 8. This study clarified that at optimum pH (pH 5.5), neodymium ions are presented as a mixture of hydrolyzed divalent complex, Nd(OH)^2+^, and trivalent free cation, Nd^3+^. Also, cobalt is presented as hydrolyzed monovalent complex, (Co(OH))^+^, and divalent free cation, Co^2+^. It is known that the hydrolyzed cations are more hydrophobic than free ions; hence, cobalt and neodymium ions are sorbed as hydrolyzed complexes and stick to the sorbent surface (Metwally et al. 2018).

#### Effect of contact time

**Fig. 9:**
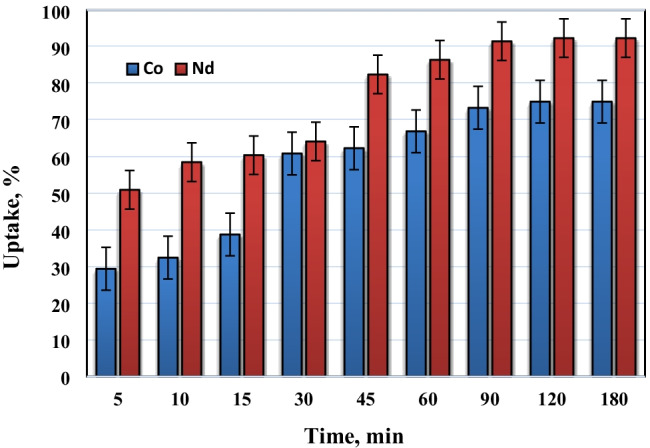
Effect of time on the sorption of 200 mg/L of Co^2+^ and Nd^3+^ by PVP–HEMA–TA at pH 5.5, room temperature, and V/m=100 mL/g

Figure 9 displays the uptake of Co^2+^ and Nd^3+^ by PVP–HEMA–TA. The outcomes illustrated that the uptake percent rises gradually until it reaches equilibrium. The sorption rate is high at the beginning then it is progressively minimized probably because of exhaustion of the available sorption sites and the growth of boundary-layer thickness. The uptake of Nd^3+^>Co^2+^ is attributed to the ionic radius; since both ions are sorbed as hydrated ions as mentioned above, and it is known that the smaller ionic radius is the largest hydrated ionic radius, therefore, the hydrated ionic radius of Co(II)>Nd(III), hence, the sorption of neodymium is the highest.

Some kinetic models were applied including pseudo-first-order and pseudo-second-order models in the non-linear forms as follows.

##### Pseudo-first-order model

Kinetic models provide data regarding sorption pathways and possible involved mechanisms. They are also imperative data for the process development and the sorption system design (Tapas and Benedicte 2020).

Pseudo-first-order kinetic model was submitted by Lagergren (1898). The rate equation of Lagergren represents the rate for the sorption from the solution which can be formulated as follows (Ghaly et al. 2018):

7 $$\frac{\mathrm{d}{q}_t}{\mathrm{d}t}={k}_n{\left({q}_e-{q}_t\right)}^n$$

where *q*_*e*_ and *q*_*t*_ are the sorbed quantity at equilibrium and time *t* (mg/g), respectively, *k*_*n*_ is the rate constant, and *n* is the sorption reaction order. If *n* equals 1, integrated form of Eq. (7) for the boundary conditions, *q*_*t*_ *=* 0 when *t= 0* and *q*_*t*_*=qt* when *t= t*, will provide the pseudo-first-order equation as follows:

8 $${q}_t={q}_e\left(1-{e}^{-{k}_1t}\right)$$

*k*_1_ is the pseudo-first-order rate constant (min^−1^). It is usually noted that kinetics belong to Lagergren equation when the sorption performs over diffusion in the interface.

**Fig. 10:**
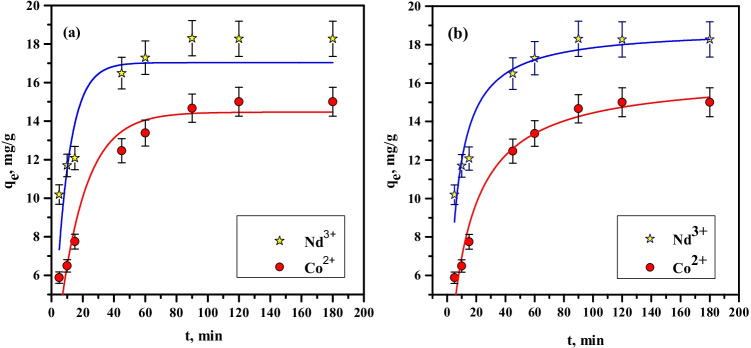
Plot of (**a**) pseudo-first-order and (**b**) pseudo-second-order models for sorption of Nd^3+^ and Co^2+^ by PVP–HEMA–TA

The plot of *q*_*t*_ against *t* and applying non-linear fitting is displayed in Fig. 10a; the pseudo-first-order rate constant, *k*_1_, and the *q*_*e*_ values were computed. Table 1 reports the values of kinetic constants along with the regression coefficient, *R*^2^, of the pseudo-first-order. However, values of the calculated *q*_*e*_ are near to those of the experimental values; this model does not designate the sorption as *R*^2^ values are low for both ions.

**Table 1:** Kinetic parameters of pseudo-first-order and pseudo-second-order models for sorption of Nd(III) and Co(II) by PVP–HEMA–TA

Ion	Pseudo-first-order model				Pseudo-second-order model		
	*q*_e_ (exp), mg/g	*q*_e_ (calc), mg/g	*k*_1_, min^−1^	*R*^2^	*q*_e_ (calc), mg/g	*k*_2_, g/mg min^−1^	*R*^2^
Nd(III)	18.27	17.03	0.11	0.72	18.8	0.0093	0.95
Co(II)	15.01	14.46	0.058	0.81	15.4	0.0044	0.96

##### Pseudo-second-order model

One main feature of this model over Lagergren is that Lagergren occasionally cannot fit well for the full range of sorption time while the pseudo-second-order model can do. The pseudo-second-order is established on the supposition that the rate-limiting step is chemisorption and estimates the performance over the full sorption range (Tapas and Benedicte 2020). The chemisorption comprises valence forces where the sorbent and sorbate exchange or share electrons and probably production of a new compound. The equilibrium sorption capacity can be computed from the model; therefore, there is theoretically no requirement to evaluate sorption equilibrium capacity from the experiment. From Eq. (7), if *n* equals 2, a pseudo-second-order equation can be gained. The differential formula of the model is formulated as

9 $$\frac{\mathrm{d}{q}_t}{\mathrm{d}t}={k}_2{\left({q}_e-{q}_t\right)}^2$$

where *k*_2_ (g/mg min^−1^) is the pseudo-second-order rate constant for the sorption process. Integrating and applying boundary conditions *q*_*t*_=0 when *t*=0 and *q*_*t*_=*q*_*t*_ when *t*=*t*, Eq. (9) becomes as follows (Kyzas et al. 2018):

10 $${q}_t=\frac{k_2{q}_e^2t}{1+{k}_2{q}_et}$$

The computed amount sorbed at equilibrium, *q*_*e*_, and the second-order rate constant, *k*_2_, can be projected from the non-linear plot of *q*_*t*_ against *t* (Fig. 10b). The results clarify that *R*^2^ values of the pseudo-second-order (Table 1) are higher than that of the pseudo-first-order model. Moreover, the computed values of *q*_*e*_ from the pseudo-second-order are more close to the practical *q*_*e*_. Therefore, the pseudo-second-order is more possible to perform the behavior over the full time range; it also shows the chemisorption process (Rao and Kashifuddin 2014). So, the pseudo-second-order is a better fit for the data than the pseudo-first-order on the sorption of cobalt and neodymium ions.

#### Equilibrium-isotherm-studies

The isotherm is still considerably from the theoretical and experimental points of view. Knowing the isotherm nature and determining its parameters makes it suitable for estimating the equilibrium quantity sorbed outside those employed in the study, particularly more diluted ones; furthermore, it is needful to scale up and design the sorption equipment. Langmuir and Freundlich equations are the greatest generally utilized sorption isotherms.

##### Extended Langmuir isotherm

**Fig. 11:**
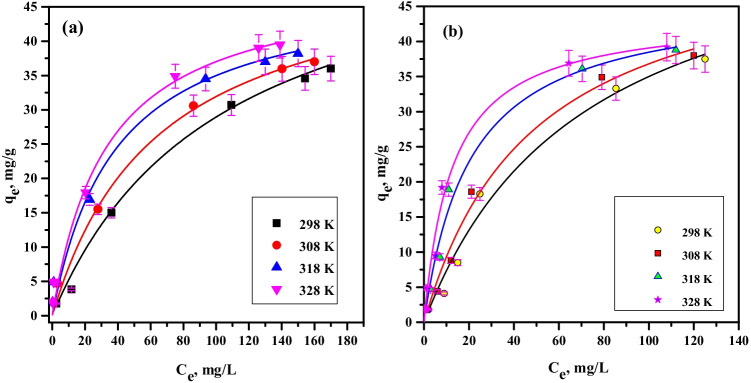
Langmuir isotherm plot for sorption of (**a**) Co^2+^ and (**b**) Nd^3+^ by PVP–HEMA–TA

Langmuir stated that all sorbent sites have the same energy and monolayer ion coverage above a homogeneous surface (Langmuir 1918). Langmuir equation in the non-linear form is clarified as follows:

11 $${q}_e={q}_m\frac{K_L{C}_e}{1+{K}_L{C}_e}$$

where *q*_*m*_ specifies the maximum sorption capacity (mg/g) and *K*_*L*_ is the sorption affinity constant (L/mg). The experimental outcomes are fitted to the sorption equation by non-linear regression fitting, as displayed in Fig. 11. Table 2 reports Langmuir parameter values; the *q*_*m*_ values of cobalt and neodymium ions are 60.8 and 64.5 mg/g at room temperature, respectively. These values demonstrate that the sorption onto PVP–HEMA–TA follows the order Nd(III)*>*Co(II) which is agreed with the order gained from the kinetic studies. The results demonstrate that Langmuir theory efficiently specifies the sorption data of cobalt and neodymium ions by PVP–HEMA–TA.

**Table 2:** Langmuir parameters for the sorption of Co(II) and Nd(III) by PVP–HEMA–TA

Temp. K	Co(II)			Nd(III)		
	*q*_m_ mg/g	*K*_L_ L/mg	*R*^2^	q_m_ mg/g	*K*_L_ L/mg	*R*^2^
298	60.8	0.008	0.97	64.5	0.014	0.97
308	65.6	0.015	0.98	70.1	0.020	0.98
318	69.5	0.025	0.98	76.2	0.049	0.98

##### Freundlich isotherm

This isotherm is established on the sorbate distribution between the solid and liquid phases at equilibrium for heterogeneous surfaces. Freundlich theory is formulated by Eq. (12).

12 $${q}_e={K}_F{C}_e^{1/n}$$

**Fig. 12:**
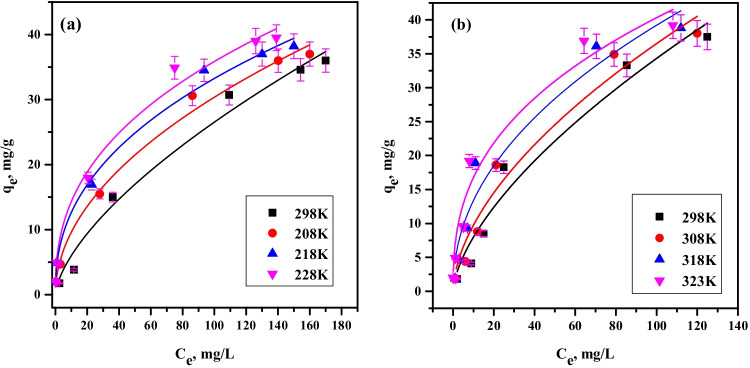
Freundlich isotherm plot for sorption of (**a**) Co^2+^ and (**b**) Nd^3+^ by PVP–HEMA–TA

*K*_*F*_ is Freundlich constant which states the sorption capacity, (mg/g)(mg/L)^1/*n*^, and *n* is dimensionless donating the surface heterogeneity. A higher *n* reveals that the system is more heterogeneous. Figure 12 displays a relation between *q*_*e*_ and *C*_*e*_; the non-linear fitting gives the values of *K*_*F*_ and *n*. The fitting results of both models (Table 3) denote that the Langmuir equation fits the data better than the Freundlich equation. Regression coefficient factors for Langmuir (*R*^2^>0.97) are higher than for Freundlich equation. The results demonstrate that all Langmuir and Freundlich parameters are temperature dependent; they rise with the rising of temperature. The increase of quantity sorbed with temperature is a strong indication that the sorption is limited by a chemisorption mechanism (Eszter and Szende 2012). In the chemisorption mechanism, the sorbed ions are strongly linked to the sorbent surface. This phenomenon is revealed by *K*_*L*_ values in Langmuir model; the stronger the interaction between sorbate and sorbent, the higher the *K*_*L*_ value.

**Table 3:** Freundlich parameters for the sorption of Co(II) and Nd(III) by PVP–HEMA–TA

Temp. K	Co(II)			Nd(III)		
	*K*_F_ (mg/g)(mg/L)^1/*n*^	1/*n*	*R*^2^	*K*_F_ (mg/g)(mg/L)^1/*n*^	1/*n*	*R*^2^
298	1.31	0.64	0.94	1.86	0.632	0.93
308	3.01	0.50	0.95	2.61	0.57	0.92
318	4.82	0.42	0.95	4.57	0.47	0.91
328	5.73	0.39	0.94	6.97	0.38	0.91

### Thermodynamic studies

The parameters of thermodynamics can be computed using Vant Hoff equation as follows:

13 $$\varDelta {G}^o=- RT\ \mathit{\ln}\ {K}_c$$

where ∆*G*^*o*^ is the standard Gibbs free energy change, *R* is the general gas constant, *T* is the absolute temperature, and *K*_*c*_ is the sorption equilibrium constant. Table 4 displays that the increase in temperature leads to an increase in the values of *K*_*c*_, thus indicating a strengthening of sorbate–sorbent interactions at high temperature. This also demonstrates that Co(II) and Nd(III) dehydrate considerably at high temperature before the sorption process and thus their sizes during the sorption are smaller yielding high *K*_*c*_ values (Naser et al. 2015). The free energy change can be represented as follows:

**Table 4:** Thermodynamic parameters for sorption of Co(II) and Nd(III) by PVP–HEMA–TA

Temp. K	*K*_c_		ΔH^o^, kJ/mol		ΔS^o^, J/mol.K		ΔG^o^, kJ/mol	
	Co(II)	Nd(III)	Co(II)	Nd(III)	Co(II)	Nd(III)	Co(II)	Nd(III)
298	0.49	0.90	40.58	56.4	130.9	187.9	−38.9	−55.9
303	0.98	1.40					−40.3	−57.8
313	1.74	3.73					−41.6	−59.7
323	2.11	6.63					−42.9	−61.5

14 $$\varDelta {G}^o=\varDelta {H}^o- T\varDelta {S}^o$$

From Eqs. (13) and (14), a relation between *ln K*_*c*_ and *∆G*^*o*^ can be obtained as follows:

15 $$\mathit{\ln}\;{K}_c=\frac{\varDelta S^o}{R}-\frac{\varDelta H^o}{RT}$$

**Fig. 13:**
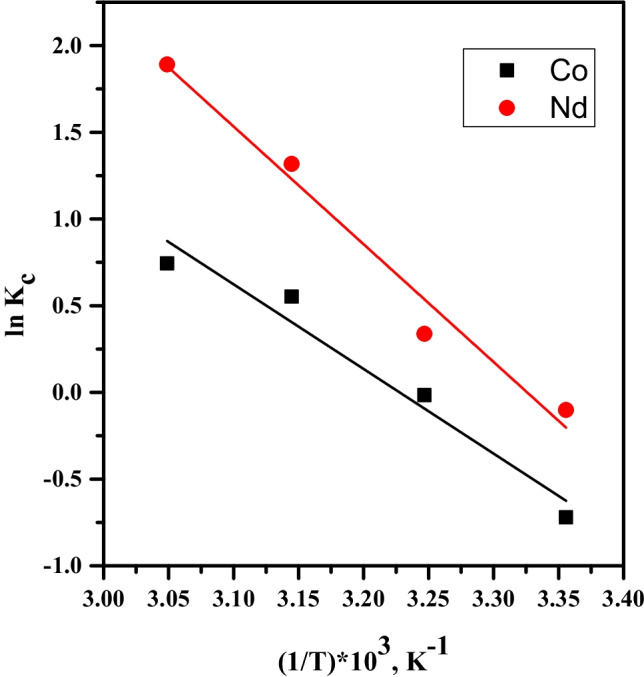
Plot of ln *K*_c_ vs. 1/*T* for sorption of Co^2+^ and Nd^3+^ by PVP–HEMA–TA

The plot of *ln K*_*c*_ versus 1/*T* gives straight lines as displayed in Fig. 13. The values of enthalpy change, Δ*H*^*o*^, and entropy change, Δ*S*^*o*^, can be calculated from the slope and intercept, respectively. The endothermic nature of the sorption process can be indicated by the positive values of enthalpy change, Δ*H*^*o*^, for both ions. The value of Δ*H*^*o*^ is greater than 20 kJ/mol; therefore, the sorption process is chemisorption (El-naggar et al. 2008). This agrees with the results obtained from the kinetic and isotherm studies. The positive values of the entropy change, Δ*S*^*o*^, show the increased randomness at the solid/solution interface with some structural changes in the sorbate and sorbent and an affinity of PVP–HEMA–TA toward Co(II) and Nd(III). The feasibility and the spontaneous nature of the sorption processes are indicated by the negative values of ∆*G*^*o*^. The data are reported in Table 4.

### Sorption mechanism

**Fig. 14:**
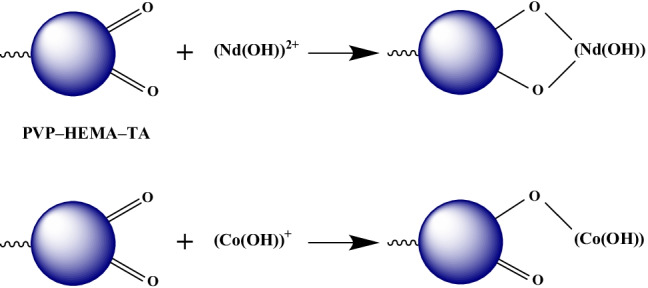
The proposed mechanism for sorption of Co(II) and Nd(III) by PVP–HEMA–TA

The sorption is controlled by a chemisorption mechanism. This is indicated by the kinetic and equilibrium studies, as mentioned above. This means that a chemical bond is formed between the sorbed metal ions and the sorbent. As discussed in the speciation studies, cobalt is presented as hydrolyzed monovalent complex, (Co(OH))^+^, and divalent free cation, Co^2+^. Also, neodymium ions are existing as a mixture of trivalent free cation, Nd^3+^, and hydrolyzed divalent complex, (Nd(OH))^2+^. It is known that the hydrolyzed cations are more hydrophobic than free ions; hence, both cobalt and neodymium ions are favorable to be sorbed as hydrolyzed complexes and bonded to the sorbent surface. The carbonyl group (C=O) is the predominant function group in PVP–HEMA–TA as illustrated in the FT-IR spectra; hence, the sorption mechanism can be proposed and displayed in Fig. 14.

### Desorption investigation

Desorption of neodymium and cobalt ions from PVP–HEMA–TA sorbent was examined using 0.5 mol/L of HNO_3_. Desorption percent, *D%*, of neodymium and cobalt ions was estimated using Eq. (6) and found to be 89.9 and 86.3%, respectively. This means that the prepared polymer composite can be reused.

### Comparison of sorption capacity of PVP–HEMA–TA with different sorbents

A comparison of monolayer capacities of neodymium and cobalt ions onto PVP–HEMA–TA with different materials attained in the literature is stated in Table 5. The data revealed that the PVP–HEMA–TA has a higher capacity than several other materials. This shows that PVP–HEMA–TA can be considered as a promised sorbent for neodymium and cobalt from solutions.

**Table 5:** Comparison of sorption capacities of neodymium and cobalt ions on different sorbents

Sorbents	Capacity, mg/g		References
	Nd(III)	Co(II)	
PVP–HEMA–TA	64.5	60.8	Present work
Cyphos@silica	5.6	NR	(Ibrahim et al. 2021)
Impregnated SiO_2_/UF composite	134.6	NR	(Naser et al. 2015)
Phosphorus functionalized adsorbent	47.5	NR	(Park and Tavlarides 2010)
Silica gel–diglycol amic acid	5.4	NR	(Ogata et al. 2015)
PP-g-(AN-co-AAc-H_2_PO_3_) polymer	NR	61.34	(Fatemeh et al. 2021)
Microcrystalline cellulose–magnesium hydroxide composite	NR	153.8	(Ruifeng et al. 2021)
Magnetic cyanoethyl chitosan beads	NR	17.9	(Qian et al. 2020)
Sediment	NR	0.97	(de Oca-Palma et al. 2021)

*NR* not reported

## Conclusion

Gamma irradiation was successfully operated for the preparation of PVP–HEMA–TA polymer. The physicochemical features of PVP–HEMA–TA were examined; the results illustrated the following aspects:

- The gelation and swelling percentages of PVP–HEMA–TA rise with rising the dose and time.
- Different TA concentrations were utilized for the preparation process, and the results illustrated that the gelation and swelling percentages decrease with raising TA concentration.
- The optimum values for the preparation process were desired at an irradiation dose of 20 kGy, 2% TA, 2% PVP, and 20% HEMA.
- Pseudo-second-order and Langmuir models fit the obtained data for sorption of Nd(III) and Co(II) ions.
- The monolayer sorption capacity of PVP–HEMA–TA is greater than several sorbents gained in the literature.
- Desorption was successfully implemented by 0.5mol/L HNO_3_.
- From the thermodynamic studies, the sorption process is spontaneous and has an endothermic nature.

The whole outcomes demonstrate that PVP–HEMA–TA is efficient and recommended for the sorption of Nd(III) and Co(II) ions from the aqueous phase.
